# A note on the rationale for estimating genealogical coancestry from molecular markers

**DOI:** 10.1186/1297-9686-43-27

**Published:** 2011-07-12

**Authors:** Miguel Ángel Toro, Luis Alberto García-Cortés, Andrés Legarra

**Affiliations:** 1 Departamento de Producción Animal, Universidad Politécnica de Madrid, 28040 Madrid, Spain; 2 Departamento de Mejora Genética, Instituto Nacional de Investigación Agraria, Ctra. de La Coruña Km 7.5, 28040 Madrid, Spain; 3 INRA, UR 631 SAGA, F-31326 Castanet Tolosan, France

## Abstract

**Background:**

Genetic relatedness or similarity between individuals is a key concept in population, quantitative and conservation genetics. When the pedigree of a population is available and assuming a founder population from which the genealogical records start, genetic relatedness between individuals can be estimated by the coancestry coefficient. If pedigree data is lacking or incomplete, estimation of the genetic similarity between individuals relies on molecular markers, using either molecular coancestry or molecular covariance. Some relationships between genealogical and molecular coancestries and covariances have already been described in the literature.

**Methods:**

We show how the expected values of the empirical measures of similarity based on molecular marker data are functions of the genealogical coancestry. From these formulas, it is easy to derive estimators of genealogical coancestry from molecular data. We include variation of allelic frequencies in the estimators.

**Results:**

The estimators are illustrated with simulated examples and with a real dataset from dairy cattle. In general, estimators are accurate and only slightly biased. From the real data set, estimators based on covariances are more compatible with genealogical coancestries than those based on molecular coancestries. A frequently used estimator based on the average of estimated coancestries produced inflated coancestries and numerical instability. The consequences of unknown gene frequencies in the founder population are briefly discussed, along with alternatives to overcome this limitation.

**Conclusions:**

Estimators of genealogical coancestry based on molecular data are easy to derive. Estimators based on molecular covariance are more accurate than those based on identity by state. A correction considering the random distribution of allelic frequencies improves accuracy of these estimators, especially for populations with very strong drift.

## Background

The concept of coancestry (or kinship) between two individuals plays a central role in practical applications of genetics. In animal breeding, coancestry coefficients are required both to estimate genetic parameters and to carry out genetic evaluations [1]. In sociobiology, they are important to make evolutionary interpretations of social behavior and to determine parameters of the biology of reproduction. In the field of animal conservation, they constitute fundamental tools to estimate inbreeding depression and to optimize genetic management in a conservation program. Several estimators of coancestries based on molecular information have been proposed, including recent estimators that are designed to deal with a large number of markers [2-7]. These estimators are based on intuitive basic identities that were explicitly shown by Cockerham [8] (and also [9]), namely, that resemblance between genotypes is a function of coancestry (identity by descent) and allelic frequencies at the base population. Interest in this subject has grown with the use of dense marker data. However, this body of literature is poorly known in the human and animal genetics communities. The aim of this work is to build estimators of genealogical coancestry from molecular coancestries and molecular covariances and to illustrate their behavior based on simulations and a real data set.

## Methods

In the following sections, *g_ik _*refers to the gene frequency value for genotypes *AA*, *Aa *and *aa*, coded *as *1, 1/2 and 0, respectively, of individual *i *at locus *k *where *i = 1, n *and *k = 1, L*. Gene frequency is half the gene content (number of copies of the reference allele A). Two animals will be referred to by indexes *i *and *j *and two loci by *k *and *l*. Allelic frequency in the base population is notated by *p*. Loci will be assumed to be neutral.

### Genealogical coancestry

In both population and quantitative genetics, the genetic relationship between individuals can be quantified by Malecot's coefficient of coancestry (or kinship) [10]. The coancestry coefficient, *f_ij_*, between individuals *i *and *j *is defined as the probability that, at a random, neutral, autosomal locus, an allele drawn randomly from individual *i *is identical by descent (IBD) to an allele drawn randomly from individual *j*. The inbreeding coefficient of an individual *i*, *F_i_*, is defined as the probability that the two alleles carried by this individual at a given locus are IBD. The inbreeding of an individual equals the coancestry between its parents. Finally, the self-coancestry *f_ii _*of an individual equals 1/2(1+*F_i_*). These coefficients can be estimated from pedigrees using the tabular method [11]. For diploid individuals, twice the coancestry coefficient is the additive relationship coefficient, which describes the ratio between the genetic covariance between individuals and the genetic variance of the base population.

### Molecular coancestry

If *n *individuals have been genotyped for one molecular marker, the molecular coancestry (or kinship), *f_Mij _*between individuals *i *and *j*, is the probability that two alleles at the locus taken at random from each individual are equal (identical by state, IBS). The coancestry concept includes the self-coancestry of an individual with itself, *f_Mii _*, in which case two alleles are drawn with replacement within individuals. Analogously, *F_Mi _*is the molecular inbreeding of individual *i*, i.e. the probability that the two alleles carried by this individual at a given locus are IBS.

By definition, *f_Mi _*= 1/2(1*+ F_Mii_*). Molecular coancestry between individuals *i *and *j *can be calculated at a given locus *k *as:

and averaged across loci as:(1)

although other alternatives could be considered [7].

### Molecular (co)variance of gene frequencies

If a set of individuals has been genotyped for several loci, we can calculate, for each individual, the variance of the gene frequencies across loci and for each pair of individuals, the covariance between two individuals, also across loci. The covariance between individuals *i *and *j *can be calculated as:(2)

where , and *L *is the number of loci.

It is important to emphasize that both molecular coancestry and molecular covariance are empirical measures of genetic similarity, and do not rely on any assumption about how the genotypes were generated. Notice that in this definition Cov_M _has to be computed over one or two individuals at a time and across loci. Therefore, it can be applied to one individual, or to individuals from different populations. Loci are considered as exchangeable (in the statistical sense), similar to how loci are treated in the context of gene dropping analysis where, instead of averaging the results over loci we can, equivalently, start the gene dropping analysis with just one locus and average over many replicates [12].

### Relationships between genealogical and molecular coancestry and molecular covariance

Here, we provide an intuitive explanation of Cockerham's [8] derivation. If the individuals are genealogically connected, the genealogical coancestry can also be defined as the molecular coancestry for 'virtual' alleles at loci that are all different in the founder population. For instance, in the gene dropping analysis [4], we start with a founder population where *n *founders have many independent loci, each with *2n *different alleles present in the founder population. If we then calculate the molecular coancestry of each pair of individuals and average over many loci, we recover precisely the same coancestry values as those calculated by, for example, the tabular or path coefficient methods.

Let us imagine now, that to each one of the 2*n *alleles at a locus in the base population, we assign a tag at random that indicates whether the allele is *A *or *a *with probability *p *and *q *= 1-*p *(because this assignment has been done at random, the genotypic frequencies *AA*, *Aa *and *aa *will be in Hardy-Weinberg equilibrium). For this locus, the molecular coancestry between two individuals will be the probability that two alleles, taken at random from each individual have the same tag (thus are IBS). This could happen in two ways: either because they have become IBD as genealogy progresses (i.e. they are copies of the same unique allele from the base population, with probability *f_ij_*), or because they are not IBD (with probability 1 - *f_ij_*) but have the same tag in the base population (with probability *p*^2 ^+ *q*^2^). Therefore, on expectation,(3)

This expression can be obtained from Equation (6) in reference [8] by summing the two events of IBS (*A *= *A *or *a *= *a*), weighted by probabilities *p *and *q*. The relationship between genealogical coancestry and molecular covariance, shown in [8], is also known from standard population genetics (e.g. [1]). Briefly, two gametes co-vary (are identical) with a probability (and correlation) *f_ij _*and thus, the covariance of the gene frequency of two individuals across loci (replicates) is (assuming the same *p *for all loci):(4)

Alternative derivations of these expressions (3) and (4) are given in the Appendix. A simple relationship exists between the expectations of molecular coancestry and molecular covariance:

From expressions (3) and (4), two different method-of-moments estimators of *f_ij _*can be obtained by reversing the formulas:(5)(6)

Expressions (3) and (5) are well known [2], whereas (6) does not seem, to our knowledge, to have been used previously.

### Accounting for variation of allelic frequencies

The above formulas refer to a scenario in which the base population has one or many independent loci with a common allelic frequency *p*. If this is not the case and *p *for individual loci is a random variable that has been sampled from a distribution with mean  and variance *Var *(*p*), taking expected values across loci, we obtain:

Then, using and , we obtain(7)(8)

The first term involving *Var *(*p*) represents a bias that results from an artificial covariance between individuals (even between unrelated ones) that is caused by variation in allele frequencies between loci. Equation (8) is derived as follows. As shown in the Appendix, the expectation of the molecular covariance between individuals *i *and *j *for a unique allele frequency *p *is

where *E*(*g_i_g_j_*) = *p*^2 ^+ *pqf_ij_*

and *E *(*g_i_*) *E *(*g_j_*) = *p*^2^.

For random allele frequencies, in addition to averaging across the sampling distribution of individuals *i *and *j *in the population (*E_population_*) one has to average also across allele frequencies (*E_loci_*), and the expression above becomes

which, after algebra, gives equation (8).

Therefore, with varying allele frequencies, estimators of genealogical coancestry based on equations (5) and (6) can be built as(9)(10)

These estimators use the same notation as expressions (5) and (6); including or not variation in allelic frequencies will depend on the context. Assuming that the allele frequencies are random draws from a Beta distribution with parameters *α *and *β*,  and *Var *(*p*) are *α*/(*α + β*) and *α*/*β *[(*α + β*)^2 ^(*α *+ *β + *1)], respectively.Thus, to extrapolate from molecular coancestry or molecular covariance to genealogical coancestry requires that the distribution of the base population allele frequencies is known, or at least its first and second moments are known. However, for practical applications, both *p *and *Var *(*p*) can be replaced by their estimates from the current population, namely(11)(12)

where(13)

Equations 5-6 and to 9-10 (using when necessary Equations 11 to 13) will be implemented in the simulations.

### Van Raden's estimators

These four methods will be compared by simulation with one of the methods proposed by Van Raden [5], which can be seen as an implementation of expression (6). In the first method proposed by Van Raden, across individual allele frequencies were computed (not necessarily using Equation (11)), and then estimators of molecular covariance were computed for each locus and then averaged over total molecular variance as follows:(14)

This method corresponds to positing a linear model where, for a hypothetical quantitative trait, the genetic value of an individual is the sum of independent marker effects; overall (i.e., due to the sum of the effects of all loci) covariance among individuals in the sample is computed first, and then standardized by the overall variance of a base population in Hardy-Weinberg equilibrium with allele frequencies equal to that observed in the sample, to arrive to additive relationships. In the second method of Van Raden (later used, for example, in [13]), estimators of genealogical coancestry are computed as in Equation (14) for each locus and then averaged, as follows:(15)

The main difference between estimators (14) and (15) is that less polymorphic loci get more credit in estimator (15). Note that Equation (15) is undefined for  equal to 0 or 1, which is not the case for Equation (14). These estimators differ slightly from the combined use of Equations (7) to (12); in Equations (14) and (15), individual allele frequencies *g_ik _*are centered with reference to allele frequencies  across individuals but within loci, whereas in Equations (7) to (12), covariances and coancestries and are computed for each pair of individuals as shown in Equations (1) and (2), i.e. individual allele frequencies *g_ik _*are centered using frequencies across loci but within individuals: . Here, loci are not exchangeable in the same sense as for equations (7) and (8), because loci with different allele frequencies in the population will contribute more or less to the covariances.

### Simulation

A population was bred from a base (founder) population of 20 individuals. One hundred or 10,000 biallelic loci representing single nucleotide polymorphism (SNP) markers, distributed over 10 chromosomes, were simulated. Loci were autosomal, unlinked, neutral, without mutation, and followed Mendelian inheritance. In the first scenario, at each locus, alleles at the founder population were sampled with a fixed probability value of *p *= 0.5. In the second scenario, at each locus, alleles were sampled with a probability taken from a flat Beta distribution *B*(1, 1). Therefore, there was Hardy-Weinberg equilibrium within loci. Ten discrete generations of 20 individuals were bred, using random mating with separate sexes, resulting in a data set of 200 individuals. We also ran some simulations with linkage between loci but the results were not much affected. Thus, we included only one example with high linkage with either 100 SNP over 1 Morgan or 10,000 SNP over 20 Morgan.

Relatedness between all pairs of individuals was estimated from the marker data using each of the four (5), (6), (9) and (10) estimators described above and those of Van Raden (14) and (15). For the second estimator of Van Raden (15), monomorphic loci were ignored because for some loci the estimated value  may be one or zero and the estimator becomes undefined. In addition, relatedness between individuals was calculated from the pedigree, using the tabular method [11] and this was considered to be the true value; this is true if there are many unlinked loci (avoiding noise due to finite sampling and co-segregation), which holds in the simulation. We also compared results to true IBD probabilities rather than pedigree coancestries. This is relevant for real situations where deviations from the average relationship exist due to linkage and finite sampling [14]. To obtain true IBD probabilities, we coded the alleles in the base population as unique alleles, with codes 1 through 2*n*.

### Real data

To illustrate the procedure on real data, a set of 1,827 French Holstein bulls genotyped with the Illumina Bovine SNP50 BeadChip for 51,325 polymorphic (minor allele frequency > 0) SNP was used. The pedigree of these bulls was traced back as far as possible, including 6,940 individuals. We used PEDIG [15] to compute the equivalent number of known generations: 4.22, and the average number of ancestors: 91.4. Estimators (5), (6), (9) (10), (14) and (15) were used to compute coancestries among the genotyped animals. Some of the computations used the preGSf90 software with methods detailed in [16].

## Results

The agreement between the molecular coancestries and molecular covariances and their expected values were checked by simulation. As for the comparison with IBD probabilities, the results were almost identical to those obtained with genealogical coancestries except for the scenario with a low number of markers. Table 1 shows the regression of the genealogical coancestry on the molecular coancestry or the molecular covariance. Very good agreement exists between expected (in estimators (5), (6), (9) and (10)) and observed values of intercept and slope when the number of SNP is very large; also, the coefficients of determination are close to 1. This occurs in the two considered situations (*p *fixed or *p *variable among loci). The coefficients of determination are low when the number of SNP is low, especially when the allele frequencies in the base population are variable.

**Table 1:** Features of the regression of genealogical coancestry *f *on molecular coancestry (*f_M_*) and molecular covariance (*Cov_M_*)

Nb SNP	Nb replicates	Regression on coancestry			Regression on covariance		
		**a**	**b**	**R^2 ^**	**a**	**b**	**R^2^**

*p *= 0.50							
100	1000	-0.66 (0.03)	1.38 (0.06)	0.69 (0.03)	0.03 (0.00)	2.77 (0.12)	0.69 (0.03)
10000	50	-0.99 (0.00)	1.99 (0.01)	1.00 (0.01)	0.00 (0.00)	3.98 (0.03)	1.00 (0.01)
Expected values					0		

*p*_i_~*Beta*(1, 1)							
100	1000	-1.01 (0.08)	1.58 (0.10)	0.52 (0.06)	-0.22 (0.04)	3.17 (0.21)	0.52 (0.06)
10000	50	-1.98 (0.02)	2.97 (0.03)	0.99 (0.02)	-0.50 (0.00)	5.95 (0.06)	0.99 (0.00)
Expected values							

Intercept (a), slope (b) and coefficient of determination (R^2^), with standard deviations across replicates, of the regression equation of genealogical coancestry *f *on molecular coancestry (*f_M_*) and molecular covariance (*Cov_M_*), based on simulated data, when the distribution of allele frequencies in the founders (*p*) is known and fixed (*p *= 0.5) or variable (*p_i _*~ Beta(1,1)).

For the simulated data, we implemented estimators of the genealogical coancestry based on molecular coancestry (equations (5) or (9)) and molecular covariance (equations (6) or (10)), using the true or estimated frequencies. In both cases (*p *fixed or random) estimates based on coancestry and covariance were almost identical and only the regression features when using  are presented in Table 2. As expected, the estimation works very well if the number of SNP is high. If it is low, the estimation of the intercept is biased upwards and the regression coefficient downwards. When the number of SNP used to estimate  decreases, the covariance between estimator and the true value decreases and the regression coefficient also decreases; the intercept increases as a direct consequence.

**Table 2:** Features of the regression of genealogical coancestry *f *on estimators

Nb SNP	Nb replicates	Distribution of allelic frequencies known			Distribution of allelic frequencies estimated from the data		
		a	b	R^2^	a	b	R^2^
p = 0.50*							
100	1000	0.03	0.69	0.69	0.09	0.63	0.69
10000	50	0.00	0.99	1.00	0.09	0.91	1.00
Expected values		0.0	1.0		0.0	1.0	

*p_i _*~ *Beta*(1, 1)**							
100	1000	0.05	0.52	0.53	0.09	0.48	0.52
10000	50	0.00	0.99	1.00	0.09	0.90	0.99
Expected values		0.0	1.0		0.0	1.0	

Intercept (a), slope (b) and coefficient of determination (R^2^), based on simulated data, when the distribution of allele frequencies in the founders is known or estimated from the data.

*Estimators (5) and (6) are used; **Estimators (9) and (10) are used

When parameters of the true distribution of allele frequencies in the founder population are not known, we replaced them by their estimates according to Equations (11) and (12). Table 2 shows that this simple method works well with respect to the goodness of fit (R^2^) but the estimates were biased (and inflated: b < 1) even for a high number of SNP. Indeed, Van Raden [5] already pointed out that base allele frequencies should be used to recover correct inbreeding coefficients. Table 3 gives the same results but for a scenario where loci are linked, with 1 (100 SNP) or 20 (10000 SNP) Morgans per gamete. Results were very similar to the unlinked situation (Table 2), although the estimation improved for the small number of markers and worsened for the high number of markers. For the situation with linkage, we also analyzed what happens if we use IBD instead of the genealogical coancestry as the true values (right hand side of Table 3). The fit is better for IBD values than for genealogical coancestry, especially with a low number of markers.

**Table 3:** Features of the regression of genealogical coancestry *f *and identity by descent on estimators

Nb SNP	Nb replicates	Genealogical coancestry			Identity by descent		
		a	b	R^2^	a	b	R^2^
p = 0.50*							
100	1000	0.09	0.55	0.60	0.09	0.68	0.74
10000	50	0.09	0.87	0.95	0.09	0.91	1.00
Expected values		0.0	1.0		0.0	1.0	

*p_i _*~ *Beta*(1, 1)							
100	1000	0.09	0.43	0.48	0.09	0.54	0.58
10000	50	0.09	0.86	0.95	0.09	0.90	0.99
Expected values		0.0	1.0		0.0	1.0	

Intercept (a), slope (b) and coefficient of determination (R^2^), based on simulated data with linkage, when the distribution of allele frequencies in the founders is estimated from the data

*Estimators (5) and (6) are used; **Estimators (9) and (10) are used

Results presented in Table 4 show that the Van Raden estimator (14) works less well than those proposed here based on molecular coancestry or molecular covariance. The reason appears to be that inferences about the distribution of allele frequencies in the founder population are less accurate when based on the average across individuals than when based on the average across loci. In fact, the results of the Van Raden estimator improve when the distribution of allele frequencies is estimated from the data of the last five generations (R^2 ^= 0.69 or 0.96 for 100 and 10000 SNP, respectively) or when the population simulated comprises four generations of 50 individuals per generation (R^2 ^= 0.53 or 0.96 for 100 and 10000 SNP, respectively). Thus, strong drift exacerbates the problem. Results from the second estimator of Van Raden (15) were almost identical to those from estimator (14).

**Table 4:** Features of the regression equation of genealogical coancestry *f *on the first estimator of Van Raden

Nb SNP	Nb replicates	Without linkage			With linkage		
		**a**	**b**	**R^2^**	**a**	**b**	**R^2^**

p = 0.50							
100	1000	0.09	0.57	0.36	0.09	0.48	0.30
10000	50	0.09	0.90	0.57	0.09	0.85	0.53
Expected values		0.0	1.0		0.0	1.0	

*p_i _*~ *Beta*(1, 1)							
100	1000	0.09	0.52	0.33	0.09	0.44	0.28
10000	50	0.09	0.90	0.59	0.09	0.90	0.58
Expected values		0.0	1.0		0.0	1.0	

Intercept (a), slope (b) and coefficient of determination (R^2^) using the first estimator of Van Raden (expression 14), based on simulated data without and with linkage, when the distribution of allele frequencies in the founders is estimated from the data

Considering all coancestries among the 1827 bulls in the real data set, Table 5 summarizes the comparisons among all estimators. The average genealogical coancestry was 0.04 and whereas estimators (5) and (6) were severely biased, estimators (9), (10) and (14) were (slightly) biased in the opposite direction, showing that, as described by Hayes et al. [17], they effectively set the current population as the base. We will refer to this later. Estimators (5) versus (6) and (9) versus (10) showed the same bias; estimators (5-9) and (6-10) were perfectly correlated, which is logical because they are linear transformations of each other. Only estimator (14) provided a variance of coancestries similar to genealogical values, although all estimators show higher variances; this can also be seen in the simulations because the regression coefficients were less than 1. Estimator (15) is unbiased, but shows low correlations with all other methods and higher variance due to numerical instability caused by low minor allele frequencies. Estimator (14) is an adequate estimator with regard to closeness to genealogical coancestries.

**Table 5:** Behaviour of estimators of coancestries (including self-coancestries) using pedigree (*f*) or molecular data for 1827 Holstein bulls

	f *	*f_M _*(1)	*Cov_M _*(2)	(5)	(9)	**(6)**	(10)	(14)	(15)
*f*	**0.11**	0.59	0.67	0.59	0.59	0.67	0.67	0.87	0.48
*f_M _*(1)	0.66	**0.04**	0.76	1	1	0.76	0.76	0.59	0.34
*Cov_M _*(2)	0.01	-0.70	**0.01**	0.76	0.76	1	1	0.73	0.41
(5)	0.23	-0.43	0.21	**0.19**	1	0.76	0.76	0.59	0.34
(9)	-0.05	-0.71	-0.06	-0.27	**0.37**	0.76	0.76	0.59	0.34
(6)	0.23	-0.43	0.22	0	0.27	**0.13**	1	0.73	0.41
(10)	-0.04	-0.70	-0.06	-0.27	0	-0.27	**0.24**	0.73	0.41
(14)	-0.04	-0.70	-0.01	-0.27	0	-0.27	0	**0.13**	0.58
(15)	-0.04	-0.70	-0.05	-0.27	0	-0.27	0	0	**0.32**

Correlations (upper triangle), variances (diagonal; divided by 100) and average differences (lower triangle; row estimator minus column estimator) between the different estimators.

*f is the genealogical coancestry calculated by the tabular method; for the other estimators, the corresponding formula in the text is indicated in parenthesis

## Discussion

Genetic marker data are widely used to estimate the relatedness between individuals. Such marker-based relatedness is valuable in many areas of research on the evolution and conservation of natural populations, for example for estimating heritabilities, estimating population sizes, minimizing inbreeding in captive populations, and studying social structures and patterns of mating.

Since the 1950's, many relatedness estimators have been proposed. However, in the last years, the use of high-density SNP genotypes in 'genomic selection' has prompted the need of a genomic coancestry matrix [5,17], more accurate than the pedigree-based one, because true coancestry will be affected by linkage and finite sampling [14], and also because pedigree-based genealogical coancestry is obliged to assume an average relationship among founders (usually 0). Van Raden [5,18] has proposed the use of molecular covariance to derive (more exact) genealogical coancestries. Because of its simplicity and computational efficiency, the use of molecular covariances has quickly become widespread [13,7], although its origin is often erroneously attributed [7,19]. In fact, the earliest reference we are aware of its use is [18]. Here, we have recalled Cockerham's original derivation [8] and have provided an equivalent derivation. This provides further proof for the prediction methods of gene content of non genotyped animals through pedigree relationships [20,21], which, in turn, are the basis for the single-step method to combine genomic and pedigree relationships [22,23].

We have also shown that, if we know the true distribution of the allelic frequencies in the founder population, it is possible to obtain very accurate estimates of genealogical coancestries from either molecular coancestries or covariances if the number of markers is high. Even if allelic frequencies in the base population are unknown, and the results are severely biased, a high correlation between the estimated and the true genealogical values is maintained.

In principle, it is possible to infer founder frequencies using either genealogical or marker-based relationships, possibly iteratively [7,21,24]. However, this is usually quite inaccurate and results in estimators that are very similar to crude population frequencies. In addition, a question remains on what is the ideal base population, which is unsolvable if no pedigree is known. In fact, using allelic frequencies in the observed population (crude means) is equivalent to defining, a population with the same gene frequencies as the observed population as the base generation, but with genotypic frequencies in Hardy-Weinberg equilibrium [17]. To change the base population, a correction based on Wright's F_st _coefficients has recently been suggested [25].

In practice, the computed matrix of coancestries (**G**) is used for two purposes. One purpose is the estimation of breeding values based on marker genotype data. In this case, if no other information is used (i.e., there is no use of pedigree-based relationships **A**), adding or removing constants from **G **is equivalent to fitting an overall (random) mean to the model for genetic values. Thus, estimates of breeding values will be simply shifted by a constant but their contrasts and selection decisions will be unaffected. In this case, the variance components need to be estimated with the same **G **and will be inflated. However, if mixing of **A **and **G **is needed, as in the single-step procedure [23,26], then the two matrices need to be compatible. In this case, bias due to selection can be a problem. A recent correction based on F_st _suggested by Powell et al. [25] has been proposed for the single-step method and has been shown to increase accuracy and remove bias of genetic evaluations [27,28]. This correction works, roughly, by fixing the biases and variances of the estimator of coancestry that can be observed, for instance, in Table 5.

For conservation purposes, most strategies work by minimizing the average coancestry [29], which can be expressed as a quadratic form **x'Gx**. The optimization of this expression is invariant to the addition (or multiplication) of any constant to **G**, unless more than one population is considered. If the **G **matrices are computed separately for each population, then they will not be compatible. If pooled current frequencies are used, then the more variable or more abundant population will be favored. Possibly, in this case, a clear definition of the allele frequencies (and thus the base population) to compute coancestries is needed.

In addition, the real data example shows that, in this data set, estimators based on molecular covariances are more similar and more compatible with those based on pedigree, than estimators based on coancestries, in particular estimator (14). We do not recommend estimator (15) because it does not agree well with genealogical coancestries, the distribution of coancestries has more variance, and it is unstable for minor allelic frequencies close to 0 and undefined for monomorphic loci. Unfortunately this estimator is recommended by some authors [19,7,13].

## Conclusions

The rationale to compare and estimate genealogical coancestries based on molecular empirical coancestries or covariances has been shown for any outbred or inbred population, and different estimators have been developed which account for variation in allele frequencies between loci. In practice, different estimators lead to similar conclusions. Estimators are easy to construct but suffer from a lack of knowledge on the distribution of allele frequencies in the base population. This is, however, not a problem for most practical applications.

## Appendix

We present here a formal derivation of relationship between genealogical and molecular coancestries and covariances. This is an alternative derivation to that of Cockerham [8] and to our knowledge it has not been shown so far. We will prove it for a population of outbred individuals and will sketch the proof for a population of inbred individuals.

### Outbred individuals

**Figure 1:**
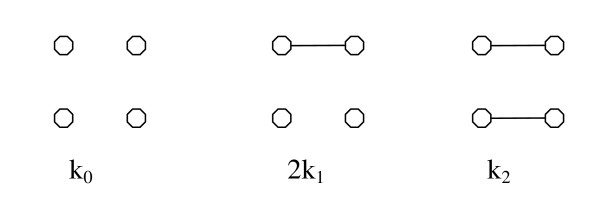
**Three modes of genetic identity-by-descent between two outbred individuals at a single locus**.

There are three ways in which a pair of relatives can share genes identical by descent (IBD) Crow and Kimura (Figure 1); *k*_0_, 2*k*_1 _and *k*_2 _are the probabilities that *x *and *y *share no genes, just one gene and both genes IBD (*k*_0 _+ 2*k*_1 _+ *k*_2 _= 1). The coancestry coefficient between two individuals is thus defined as:

The joint genotypic distribution of non-inbred relatives *i *and *j *is well known (see for example [30]), as shown in Table 6. The expected value of the molecular coancestry averaged over the nine rows will be

**Table 6:** Joint genotypic distribution of non-inbred relatives *i *and *j*

*G_i_*	*G_j_*	*f_M_*	*g_i_*	*g_j_*	Frequency
*AA*	*AA*	1	1	1	*k*_0_*p*^4 ^+ 2*k*_1_*p*^3^+ *k*_2_*p*^2^
*AA*	*Aa*	0.5	1	0.5	*k*_0_2*p*^3^*q *+ 2*k*_1_*p*^2^*q*
*Aa*	*AA*	0.5	0.5	1	*k*_0_2*p*^3^*q *+ 2*k*_1_*p*^2^*q*
*AA*	*aa*	1	1	0	*k*_0_2*p*^2^*q*^2^
*aa*	*AA*	0	0	1	*k*_0_2*p*^2^*q*^2^
*Aa*	*Aa*	0.5	0.5	0.5	*k*_0_4*p*^2^*q*^2 ^+ 2*k*_1_*pq *+ *k*_2_2*pq*
*Aa*	*aa*	0.5	0.5	0	*k*_0_2*pq*^3 ^+ 2*k*_1_*pq*^2^
*aa*	*Aa*	0.5	0	0.5	*k*_0_2*pq*^3 ^+ 2*k*_1_*pq*^2^
*aa*	*aa*	1	0	0	*k*_0_*q*^4 ^+ 2*k*_1_*q*^3 ^+ *k*_2_*q*^2^

After some algebra,

The expected value of the molecular coancestry averaged over the nine rows will be, given that *E*(*g_i_*) = *E*(*g_j_*) = *p*,

### Inbred individuals

**Figure 2:**
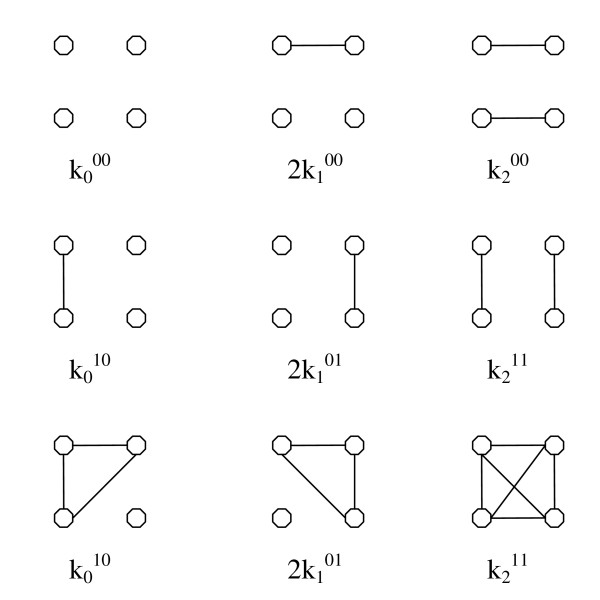
**Nine ways in which a pair of relatives can share genes identical by descent**.

When either relative may be inbred, we need nine ways in which a pair of relatives can share genes identical by descent [31] (Figure 2). The following relationships hold:

The joint genotypic distribution of non-inbred relatives *i *and *j *when either relative may be inbred is also well known (Table 7). First we need to define nine ways in which a pair of relatives can share genes identical by descent and the corresponding k-coefficients.

**Table 7:** Joint genotypic distribution of inbred relatives *i *and *j*

*G_i_*	*G_j _*	*f_M_*	*g_i_*	*g_j_*	Frequency
*AA*	*AA*	1.	1	1	
*AA*	*Aa*	0.5	1	0.5	
*Aa*	*AA*	0.5	0.5	1	
*AA*	*aa*	0.	1	0	
*aa*	*AA*	0	0	1	
*Aa*	*Aa*	0.5	0.5	0.5	
*Aa*	*aa*	0.5	0.5	0	
*aa*	*Aa*	0.5	0	0.5	
*aa*	*aa*	1.	0	0	

After algebra, we arrive to the same expressions as above for and . Note that the proof of Cockerham [8] is general and applies to either outbred or inbred populations.

## Competing interests

The authors declare that they have no competing interests.

## Authors' contributions

MT derived the theory with help from LAGC and AL. MT and LAGC ran the simulations and AL the real data example. All authors participated in the discussion and wrote the final manuscript.
